# Predicting Outcome after Acute Severe Ulcerative Colitis: A Contemporary Review and Areas for Future Research

**DOI:** 10.3390/jcm13154509

**Published:** 2024-08-01

**Authors:** Sudheer Kumar Vuyyuru, Olga Maria Nardone, Vipul Jairath

**Affiliations:** 1 Departments of Medicine, Division of Gastroenterology, Schulich School of Medicine & Dentistry, Western University, London, ON N6A 5C1, Canada; 2 Gastroenterology, Department of Public Health, University Federico II of Naples, 80131 Naples, Italy; 3 Division of Epidemiology and Biostatistics, Western University, London, ON N6A 5C1, Canada; 4 Lawson Health Research Institute, London, ON N6A 3K7, Canada

**Keywords:** steroid-refractory, rescue therapy, subtotal colectomy, ulcerative colitis

## Abstract

Acute Severe Ulcerative Colitis (ASUC) is a severe form of ulcerative colitis relapse which requires hospitalization and intensive medical intervention to avoid colectomy. The timely recognition of patients at risk of corticosteroid failure and the early initiation of medical rescue therapy are paramount in the management of ASUC. The choice of medical rescue therapy is influenced by multiple factors, especially patient’s prior treatment history. This decision should involve the patient and ideally a multidisciplinary team of healthcare professionals, including gastroenterologists, radiologists, surgeons and enterostomal therapists. Although several predictive models have been developed to predict corticosteroid failure in ASUC, there is no single validated tool that is universally utilized. At present, infliximab and cyclosporine are the only agents systematically evaluated and recommended for medical rescue therapy, with recent reports of off-label utilization of tofacitinib and upadacitinib in small case series. The available evidence regarding the efficacy and safety of these oral small molecules for ASUC is insufficient to provide definitive recommendations. Early decision-making to assess the response to medical rescue therapy is essential, and the decision to pursue surgery in the case of treatment failure should not be delayed.

## 1. Introduction

Ulcerative colitis (UC) is a type of inflammatory bowel disease (IBD) disease limited to the large intestine, affecting approximately 5 million people globally, with increasing incidence in developing countries [1]. Approximately 25% of patients with UC experience severe flare-up requiring hospitalization and intravenous (IV) corticosteroids during their disease course [2]. With the availability of novel advanced biologic therapies and optimization of management algorithms, the hospitalization rate attributable to acute flare-up appears to have reduced. However, with shifting patterns in the epidemiology of UC, [3] there has been a rise in hospitalizations in newly industrialized countries [4]. This observation could, in part, be attributed to increasing incidence of UC alongside the limited availability of advanced therapies in these countries [5]. An episode of acute severe UC (ASUC) is the severe form of UC relapse and is a medical emergency with overall mortality of 1%, with high mortality in older individuals [6]. Since Truelove and Witts demonstrated the benefit of IV corticosteroids in a randomized controlled trial (RCT) in 1955, corticosteroids remain the first treatment of choice in patients with ASUC [7]. More than one third of patients with ASUC do not respond to IV corticosteroids and require medical rescue therapy. Surgical rescue therapy with emergency colectomy may be needed in medically refractory cases and in cases of complications such as toxic megacolon or colonic perforation [8]. Approximately 20% of patients admitted with ASUC require a subtotal colectomy with temporary end ileostomy on their first admission, despite medical rescue therapy followed by ileal pouch anal anastomosis later on [9]. The risk of colectomy increases after subsequent episodes of ASUC [2,10]. Several classes of biologics and oral small molecules have been evaluated in clinical trials and were approved for the management of moderate to severe UC, although these trials did not include patients with ASUC. At present, infliximab and cyclosporine are the only agents systematically evaluated and recommended as medical rescue therapy in steroid-refractory ASUC [11]. Timely decision-making is fundamental, and excessive delays in proceeding to surgery in cases of failure of medical therapy can lead to increased postoperative morbidity and mortality [12]. This underscores the importance of being able to reliably predict response to medical therapy early in the course of ASUC to facilitate timely decision-making. Several clinical, biochemical and endoscopic scoring indices and composite scores with combinations of these factors have been shown to be useful for predicting response to corticosteroid therapy, medical rescue therapy and subsequent colectomy in patients admitted with ASUC. Unfortunately, there is no single universally accepted index which can accurately predict response to corticosteroid therapy. The Oxford criteria were developed more than two decades ago in the pre-biologic era and remain the most commonly used index to predict response to corticosteroids [13]. Whilst newer indices have been developed in more recent times, their operating properties have not been fully validated, and there are no indices available for accurately predicting failure of medical rescue therapy. In this review, we discuss general management principles and existing indices for predicting corticosteroid and medical rescue therapy failure and highlight areas that need to be further explored to improve management of ASUC.

## 2. Medical Management of ASUC

The criteria proposed by Truelove and Witts (TW) more than 70 years ago remain the most commonly used for initial risk stratification of patients with ASUC [7]. These criteria were based on clinical symptoms (including the presence of ≥ 6 bloody stools/day) and laboratory features of systemic inflammation (at least one of the following signs of systemic inflammation: heart rate > 90 beats/min, temperature > 37.8 °C, hemoglobin < 105 g/L and/or erythrocyte sedimentation rate (ESR) >30 mm/h). These criteria were later modified to include elevated C-reactive protein (CRP) >30 mg/L instead of raised ESR [14]. TW criteria are generally not used for assessment of response to therapy as there are no validated criteria for defining response. TW criteria were later modified by Lichtiger et al., with eight variables including diarrhea (number of daily stools), nocturnal stools, visible blood in stool, fecal incontinence, abdominal pain/cramping, general well-being, abdominal tenderness and need for antidiarrheals [15]. The Lichtiger index ranges from 0 to 21, and some clinical trials have defined ASUC as a Lichtiger index score of more than 10 [16,17].

Patients satisfying criteria for ASUC should be hospitalized and standardized management approach to prevent complications and avoid colectomy should be followed. IV corticosteroids (hydrocortisone 300–400 mg/day or methylprednisolone 60 mg/day) should be initiated as a first-line therapy. Corticosteroids have significantly reduced mortality from 30% to 1% [6]. Nonetheless, approximately one third of patients with ASUC fail to respond to corticosteroids [18]. Parenteral nutrition is generally not recommended unless enteral nutrition is clearly contraindicated [19]. Moreover, enteral nutrition has been shown to be associated with less adverse events and greater improvement in serum albumin levels compared to parenteral nutrition [19]. Thromboprophylaxis should be considered for all patients of ASUC unless there is a clear contraindication [20]. *C. difficile* infection, or other enteric pathogen, should be ruled out, and appropriate antibiotics should be administered if positive. Routine use of antibiotics is not recommended. Immunohistochemistry for Cytomegalovirus (CMV) should be performed on rectal mucosal biopsies in suspected cases, especially in corticosteroid-refractory cases and presence of deep ulcers on sigmoidoscopy or rectoscopy, and appropriately treated with antiviral therapy [21]. Routine use of CMV polymerase chain reaction analysis of peripheral blood and colonic biopsy tissues is not recommended as sensitivity and specificity are suboptimal [22]. However, some studies showed that quantitative analysis of CMV viral deoxyribonucleic acid (DNA) copies in colonic mucosal tissue samples is associated with adverse outcomes in ASUC [23].

Patients who fail to respond to IV corticosteroids require either medical or surgical rescue therapy. Either cyclosporine or infliximab can be considered for medical rescue therapy. The efficacy of cyclosporine (4 mg/kg IV) in ASUC was first demonstrated in a small study of 20 participants with ASCU who were refractory to coticosteroids; out of 11 patients who received cyclosporine, 82% had significant improvement in modified TW severity index compared to none of the 9 patients in the placebo arm [24]. A subsequent study demonstrated the similar efficacy of 2 mg/kg cyclosporine compared to 4 mg/kg dosing [25]. Two head-to-head landmark RCTs comparing infliximab and cyclosporine showed the similar efficacy of both agents (Table 1).

In the CySIF trial, patients with ASUC not responding to IV corticosteroids were randomized to receive IV cyclosporin or infliximab followed by azathioprine in both groups [16]. In this study, there was no statistically significant difference between both interventions in treatment failure at day 98. Similarly, there was no difference in colectomy rate and serious adverse events between both groups. Subsequently, a pragmatic mixed methods trial (CONSTRUCT) compared quality-adjusted survival between cyclosporine and infliximab in patients with ASUC who had failed to respond to IV corticosteroids [26]. There was no significant difference between both agents for the primary outcome, nor for colectomy during hospital admission, at 3 months and 12 months (Table 1). A meta-analysis of 16 studies including data from RCTs also did not show any difference between these two agents [27]. A major concern for usage of cyclosporine is nephrotoxicity and neurotoxicity, especially when associated with hypercholesterolemia and hypomagnesemia. Therefore, long-term use of cyclosporine is not preferred by clinicians. IFX is generally preferred over cyclosporin despite evidence showing no difference in efficacy and safety between these two agents because of the ease of administration of IFX and need for intensive monitoring for cyclosporine. Tacrolimus is another calcineurin inhibitor that was evaluated in a few observational studies for management of ASUC. In a systematic review, tacrolimus demonstrated superior efficacy compared to a placebo for steroid-refractory moderate to severe UC [28]. However, the data in ASUC limited. An observational study demonstrated that tacrolimus was effective in patients with steroid-refractory ASUC and IV tacrolimus is considered better than oral tacrolimus [28,29,30]. However, tacrolimus is largely not used in because of similar practical issues to cyclosporine.

Delaying decision for colectomy is associated with worse outcomes [31]. Therefore, the decision to use medical rescue therapy after failure of corticosteroids and decision to proceed to colectomy after failure of medical rescue therapy should be considered within 5–7 days of initiation of rescue therapy. In the presence of complications such as perforation, toxic megacolon refractory to medical therapy, and excessive bleeding causing hemodynamic instability, surgery should not be delayed. Several indices help in predicting steroid failure and need for medical recue therapy early in the treatment algorithm, helping in making these crucial decisions.

## 3. Predictors of Response to Corticosteroids

**Figure 1:**
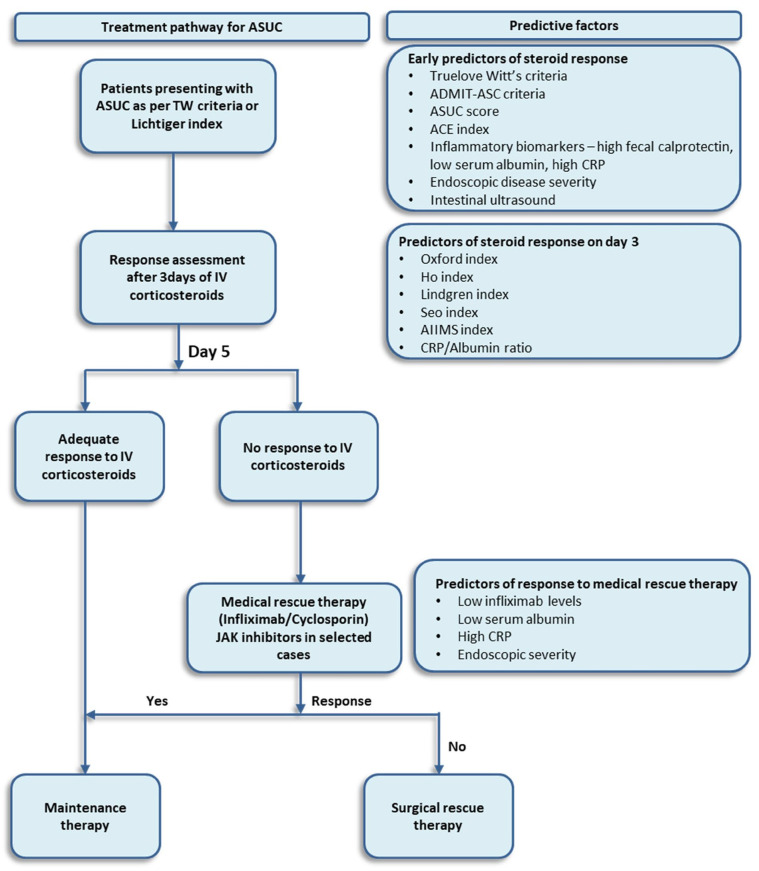
Treatment pathway for the management of ASUC and factors predicting response to medical therapy.

Response to corticosteroid therapy is generally assessed after 3 to 5 days before proceeding to rescue therapy. Prolonged use of corticosteroids beyond 7 days has been shown to be associated with high morbidity [32], and dose higher than recommended are not superior to standard doses [18,33]. Several prognostic indices, incorporating clinical and biochemical parameters and endoscopic assessment, have been developed to predict the failure of corticosteroid therapy and the need for subsequent colectomy (Table 2) (Figure 1). Despite the availability of objective biomarkers and endoscopic disease assessment tools, stool frequency and the presence of blood in stool remain valuable and commonly used measures to monitor response to medical therapy and were incorporated in most indices. A recent study reported that absence of rectal bleeding at the time of discharge predicted corticosteroid-free clinical remission and endoscopic remission after discharge [34].

Newer predictive indices predominantly incorporated objective parameters like endoscopic features. The following are the various important composite indices that were developed in chronological order.

*Truelove Witts’s criteria*: TW criteria were initially developed in 1955 to classify patients with UC according to severity of disease activity. It was later recognized that these criteria strongly predict colectomy. The risk of colectomy increases with number of additional criteria on admission in addition to at least six bloody stools per day (1 criterion: 8.5%; ≥3 criteria: 48%), indicating a proportional relationship between inflammatory burden and severity of disease activity [2].

*Oxford criteria*: These were developed by Travis and colleagues in 1996. Moreover, patients with complete response (< or = 3 stools on day 7, without visible blood) had lower chance of colectomy (5%) at a median follow up of 9 months compared to partial responders (>3 stools or visible blood on day 7, but no colectomy) (40%). Although the Oxford criteria were developed to predict need for surgery before the advent of biologic therapies, they remain the most frequently used index because of their simplicity. Even in the post-biologic era, the Oxford criteria have demonstrated a reasonably good performance characteristics (sensitivity of 62%, specificity of 64% and positive predictive value of 56%) in predicting response to corticosteroid therapy [35].

*Seo index*: This index was developed in 1992 using five parameters, namely bloody stool (little or none = 0, present = 1), bowel movements (≤4 = 1, 5–7 = 2, ≥8 = 3), ESR (mm/h), Hb (g/dL) and serum albumin (g/dL) [36]. Initially, this was developed as a UC disease activity assessment tool with Index values <150, 150 to 220 and >220 nearly corresponding to mild, moderate, and severe disease, respectively, in TW classification. Subsequently, performance of the Seo index was evaluated to predict colectomy in hospitalized patients with ASUC [37]. A colectomy rate of 34%, 39%, 44% and 45% was observed with a scores of 180, 190, 200 and 210 at admission, respectively. Furthermore, 97% of patients with a score less than 180 after one week of medical therapy did not require colectomy, and a score greater than 210 predicted colectomy in 61%. However, this score was also developed in the prebiologic era and was not validated subsequently.

*Lindgren index*: In the index developed by Lindgren and colleagues in 1998, CRP ≥25 mg/L and stool frequency >4/day on day 3 of corticosteroids were strongest predictors of colectomy in patients with ASUC (N = 97) [38]. A score of >8 on day 3 of IV corticosteroids was associated with colectomy in 72% of patients within 30 days.

*Ho index*: Ho and colleagues developed a risk score in 2004 using SF, transverse colonic diameter (≥5.5cm) on abdominal x-ray and hypoalbuminemia (<30 gm/L) on day 3 to predict failure of corticosteroids in patients with ASUC (N = 167). A numerical risk score ranging from 0 to 9 was developed based on these variables. Patients with scores of 0–1, 2–3 and ≥4 had a medical therapy failure rate of 11%, 43% and 85%, respectively.

*CRP/Albumin ratio (CAR)*: In a single-center retrospective study on 124 ASUC patients, day 3 CAR was a more accurate marker of steroid responsiveness than day 3 CRP or day 3 albumin alone [39]. CAR of >0.85 on day 3 predicted steroid response in 70%. Specificity of CAR further improved after combining stool frequency.

*AIIMS index*: The Ulcerative Colitis Endoscopic Index of Severity (UCEIS) was developed to assess endoscopic severity of UC and has components of vascular pattern, mucosal bleeding, erosions and ulcers with score ranging from 0 to 8 [40]. In a retrospective study of 89 patients of ASUC, 79% (N= 11/14) of patients with a UCEIS score of ≥ 7 required medical rescue therapy [41]. A new index without use of clinical factors was developed based on UCEIS and FCP (AIIMS index). All patients with a UCEIS score of >6 on day 1 and a fecal calprotectin level of >1000 µg/g of stool on day 3 required colectomy during index admission [35]. Either of the features had a sensitivity of 81%, specificity of 68% and positive predictive value of 65%. The score has been prospectively validated by the same group of authors demonstrated similar performance characteristics and better specificity (100% vs. 83%, *p* = 0.04) and positive predictive value (100% vs. 64%, *p* = 0.03) than the Oxford criteria in predicting steroid failure in ASUC [42]. However, the sensitivities of the AIIMS index (53%) and Oxford criteria (47%) were low.

*ACE index*: A simple UC severity score was derived from CRP ≥ 50 mg/L (0 or 1), albumin ≤ 30 g/L (0 or 1) and increased endoscopic severity on physician’s global assessment (0 or 1), with score ranging from 0 to 3. Subsequently the ACE index was validated by using data from CONSTRUCT trial, but the performance indices were slightly inferior compared to derivative cohort.

*ADMIT-ASC index*: An early predictive model composed of only objective parameters (serum albumin, CRP and UCEIS) was developed recently and was validated in two independent cohorts. ADMIT-ASC index comprises of CRP of ≥100 mg/L (1 point), serum albumin of ≤25 g/L (1 point) and UCEIS score of ≥4 (1 point) or ≥7 (2 points) with a total score ranging from 0 to 4. Even though a score of 0 predicted 100% steroid response and a score of 4 predicted 100% non-response to corticosteroids, very few patients had a score of either 0 or 4. A score of 3 or greater on the day of admission had a positive predictive value of 80–84% for predicting corticosteroid failure in validation cohorts [43].

ASUC score: A group of authors from Australia developed the ASUC score with variables of serum albumin ≤30 g/L, steroid use at admission and UCEIS ≥ 7 (1 point to each variable) based on data from 194 episodes of ASUC in 153 patients [44]. A total of 92% of patients with a score of ≥2 had failed IV corticosteroid therapy.

**Table 2:** Predictive indices for corticosteroid failure in patients hospitalized with acute severe ulcerative colitis.

Name of Score, Year	Design of the Study	Number of Participants	Components of Score	Cut Off	Day of Assessment Following Initiation of IV Corticosteroids	Performance Characteristics
Oxford criteria, [13]. 1996	Prospective	49	SF > 8/day or SF 3 to 8/day with CRP > 45 mg/L	-	Day 3	PPV = 85%
Lindgren index, [38]. 1998	Retrospective	97	CRP (mg/L) × 0.14 + stool frequency	>8	Day 3	Sensitivity (76.4%) Specificity (80.7%) PPV (72%)
Seo index, [37]. 2002	Retrospective	127	60 x number of bloody stool + 13 × bowel movements + 0.5 × ESR (mm/h) − 4 × hemoglobin (g/dL) − 15 × albumin (g/dL) + 200	>200	After 1 weeks of medical therapy	PPV (83%)
Ho index, [45]. 2004	Retrospective	167	Stool frequency, colonic dilatation, serum albumin levels	≥4	Day 3	Sensitivity (85%) Specificity (75%) PPV (77.2%)
AIIMS index, [35]. 2017	Prospective	45	UCEIS > 6 and FCAL >1000 µg/g	-	On admission: UCEIS Day 3: FCAL	Sensitivity (29%) Specificity (100%) PPV (100%)
CRP/Albumin ratio, [39]. 2018	Retrospective	124	CRP Serum Albumin	>0.85	Day 3	Sensitivity 70% Specificity 76%
ACE index, [39,46] 2020	Retrospective	124	CRP ≥50 mg/L (1 point), serum albumin ≤30 g/L (1 point), severe disease on endoscopic assessment (1 point)	3	On admission	Sensitivity (73.5%) Specificity (89.7%) PPV (78.1%) NPV (87.1%)
ASUC score, [44]. 2020	Retrospective	194 episodes in 153 patients	S. albumin ≤ 30 g/L, Steroid use at admission, and UCEIS ≥ 7	≥2	NA	Sensitivity 45.6%, Specificity 96.7%, PPV 92.3%, NPV 67.4%, Accuracy 73%
ADMIT-ASC score, [43]. 2022	Retrospective	Discovery cohort −117 Validation cohort −190	CRP ≥ 100 mg/L (1 point), serum albumin ≤ 25 g/L (1 point), UCEIS ≥4 (1 point) or ≥7 (2 points)	≥3	On admission	Sensitivity (32%) Specificity (96%) PPV (84%)

ACE, albumin, CRP, and endoscopy; CRP, C-reactive protein; FCAL, fecal calprotectin; IV, intravenous; NPV, negative predictive value; PPV, positive predictive value; SF: stool frequency; UCEIS, Ulcerative Colitis Endoscopic Index of Severity.

## 4. Predictors of Response to Medical Rescue Therapy

One in five patients of ASUC receiving medical rescue after corticosteroid failure require colectomy during same admission. With advent of biologic usage and advancements in monitoring, the colectomy rate has decreased from 29% to 15% (*p* = 0.033) and readmission rates from 35% to 12% (*p* = 0.0017) in the post-biologic era [43]. Low infliximab levels have been hypothesized to be one of the important factors that determine response to infliximab. Several factors that influence infliximab levels such as high mucosal and systemic levels of tumor necrosis factor (TNF), which can act as an “antigen sink” [47], excessive intestinal losses because of increased gut permeability secondary to mucosal ulceration and low serum albumin levels [48]. Some studies have explored the performance of already existing indices for predicting corticosteroid failure in predicting response to medical rescue therapy. In a study based on a United Kingdom (UK) national IBD audit, patients satisfying criteria for the high-risk category according to Oxford criteria (either SF > 8 or SF of 3–8 with CRP > 45 mg/L) and Ho index (score between 4–9) were more likely to undergo fail medical rescue therapy [49]. Similarly, some studies have shown that age >40 years and factors suggestive of inflammatory burden such as high CRP, low serum albumin and severe endoscopic disease activity at the time of infliximab initiation have been shown to be predictive of failure of medical rescue therapy [9,50]. An accurate predictive model for identifying patients who are likely to fail medical rescue therapy is an unmet need and future research should focus on developing these indices.

## 5. Predictors of Colectomy

Long-term colectomy rates (>1 year) after medical salvage therapy remain high at 30–50% despite the availability of biological therapy and improved medical management protocols [9]. Response to initial corticosteroid therapy during admission can determine the risk of colectomy after discharge from hospital. In the Oxford cohort, patients who had complete responders (SF ≤ 3 on day 7 with no blood) had a 5% chance of colectomy, whereas incomplete responders (SF > 3 or visible blood on day 7 with no colectomy in index admission) had 40% chance of colectomy at a median duration of follow up of nine months. In early responders to corticosteroids, the relapse-free rate in corticosteroid responders also remains as low as 58% [51]. In a retrospective observational study, immunomodulator use prior to admission with ASUC was associated with a medium-term colectomy rate increased by three-fold at 1 year [52]. Data analyzed from 63 patients of ASUC showed a combination of serum albumin ≤2.5 g/dL at admission, and band neutrophil count ≥ 13% at the time of initiation of infliximab had a 100% positive predictive value for 90-day colectomy [53]. Another group of authors developed a point scoring system ranging from 0 to 4 based on prior biologic exposure (1 point for one class of biologics and 2 points for multiple classes of biologics), requirement of salvage therapy during ASUC episode (1 point) and age <40 years (1 point) to predict colectomy within one year. A score of 0 was associated with no colectomy and 4 was associated 75% chance of colectomy within one year of ASUC episode [54]. Similarly, another study from French group of researchers reported a scoring system based on previous anti-TNF or thiopurines, serum albumin <30 g/L, Clostridium difficile infection and CRP > 30 mg/L, on admission with one point for each item [55]. A cumulative risk of colectomy within the next 12 months was 100% in patients with a score of 4 compared to 0% in patients with a score of 0. Endoscopic disease activity has also been shown to be a predictor of colectomy. A Mayo endoscopic score of 3 predicted colectomy within 12 months with 91% sensitivity, 48% specificity, a PPV of 37% and a NPV of 94%. In the same meta-analysis, authors reported that a CRP/albumin ratio of >0.37 at discharge predicted future colectomy (sensitivity—80%, specificity—62%, positive predictive value—42% and a negative predictive value—90%) with an AUC of 0.73. Similarly, a cut-off of <0.32 predicted avoidance of colectomy in the first 12 months (sensitivity—90%, specificity—55%) [56]. In a retrospective study, authors examined performance of various existing predictive models in predicting long-term colectomy rate at 12 months in patients with ASUC following discharge. The authors found that CRP/lymphocyte ratio and CRP/albumin ratio on day 3 performed better than the Ho index, Mayo score, Oxford criteria and Lindgren index in predicting colectomy in the 12 months [57]. In the recent post hoc analysis of TRIUMPH trial, 75% (6/8) of patients achieving a rectal bleeding (RB) sub-score of Mayo clinic score of 0 on day 7 experienced clinical remission at week 26 compared to 18% (2/11) in those with an RB sub-score of 1 [34].

## 6. Unresolved Challenges in ASUC Management

Despite advancements in management of ASUC and improve mortality over the years, there are many unanswered questions that need to be further explored [58].

### 6.1. Accelerated/Intensified Dosing Schedule of Infliximab

Accelerated infliximab regimen (5 mg/kg IV at weeks 0, 2 and 6, followed by every 8 weeks) with higher doses (10 mg/kg), is often considered by clinicians, especially in patients with low serum albumin levels. However, evidence supporting this practice is equivocal. Several observational studies have reported significantly low colectomy rates with accelerated infliximab. In a retrospective study, 35% of patients receiving a single infusion of infliximab underwent colectomy compared with 5% of patients who had received more than one infusion, which was statically significant (*p* = 0.001) [50]. Similarly, another study demonstrated the benefit of intensified infliximab compared with historical controls (6.7% vs. 40%, *p* = 0.039) over a short time. However, notably longer-term colectomy rates were similar between standard and accelerated dosing regimens [59]. Conversely, a pooled analysis of 10 studies with a total of 705 patients of whom 308 received intensified infliximab regimen showed no difference in either short-term (17% vs. 14.5%) or long-term (25% vs. 30.7%) colectomy rates with the intensified regimen compared to the standard dosing schedule. Moreover, there was no significant difference in complication rates [60]. In a recent open-label randomized trial (NCT02770040) conducted in 13 Australian centers, steroid-refractory ASUC patients (N = 138) were randomized to receive a first dose of 10 mg/kg or 5 mg/kg IFX in a 1:2 ratio [61]. Patients in the 5 mg/kg group were re-randomized in a 1:1 ratio to standard or accelerated induction groups. Patients in the 10 mg/kg group received a second dose at day 7 or earlier at the time of non-response. There was no statistically significant difference in clinical response at day 7 between the 10 mg/kg and 5 mg/kg groups (65% [30/46] vs. 61% [56/92]). In the 5 mg/kg group, response at day 7 was numerically lower in patients with low serum albumin (<25 gm/L) compared to those with high serum albumin (≥25 gm/L) (47% vs. 68%, *p* = 0.07). A similar difference was not observed in the 10mg/kg group when stratified based on serum albumin levels. However, patients receiving intensified or accelerated induction achieved clinical and biochemical remission earlier compared to standard induction, but there was no difference in outcomes at three months. Therefore, accelerated or intensified dosing of infliximab can be considered in a select group of patients, especially in patients with low serum albumin levels (≤25 gm/L) and high inflammatory burden. Future studies exploring pharmacokinetic models to personalize dosing based on baseline clearance, as well as comparative trials between clearance-based and standard dosing regimens, are highly awaited.

### 6.2. Small Molecules for Medical Rescue Therapy

Oral Janus Kinase (JAK) inhibitors such as tofacitinib and upadacitinib have been approved for the management of moderate to severe UC following results of well-designed phase 3 induction and maintenance clinical trials [62,63]. There has been a concern of increased thrombotic and cardiovascular adverse events with tofacitinib in patients with rheumatoid, especially in patients over the age of 50 with known cardiovascular risk factors [64]. Consequently, a black box warning has been issued by the Food and Drug Administration (FDA) for all JAK inhibitors. The role of JAK inhibitors in the management of ASUC has been recently explored in small case series and pilot studies. Several characteristics of oral JAK inhibitors make them an attractive choice for medical rescue therapy in patients with ASUC. Being small molecules, they are readily absorbed, and with rapid onset of action, a quick symptomatic improvement can be seen [65]. Second, small molecules are less susceptible to intestinal loss, unlike biologics such as infliximab. Third, tofacitinib and upadacitinib have been shown to be effective in patients with prior biologic exposure [66]. Recently a few reports have described off-label use of tofacitinib in patients with ASUC who did not respond to corticosteroid therapy [67,68]. In an interim analysis from recent phase 4 prospective interventional trial conducted across five Canadian hospitals (NCT04925973) recruited ASUC patients refractory to 3 days of IV corticosteroids; 24 patients with ASUC received tofacitinib 10 mg twice daily [69]. Day 7 clinical response was achieved in 58.3% (14/24) patients, and at six months, 45.8% (11/24) patients remained on tofacitinib. In another recent pilot RCT, 104 patients with ASUC were randomized to receive tofacitinib 10 mg three times daily for 7 days while continuing IV corticosteroids [70]. At day 7, statistically significant proportion of patients receiving tofacitinib achieved response (83.01% [44/53] vs. 58.82% [30/51], *p* = 0.007) compared to patients receiving placebo. Notably, one patient receiving tofacitinib developed dural venous sinus thrombosis. There is limited evidence exploring the efficacy of upadacitinib for the management of ASUC. A recent case series has reported successful treatment with upadacitinib in steroid-refractory patients [71,72,73]. Oral JAK inhibitors are potential and effective treatment options for steroid-refractory ASUC and selected patients in the absence of cardiovascular risk factors.

### 6.3. Biologics for Maintenance of Remission Following IV Cyclosporine

Patients with ASUC who respond to cyclosporine rescue therapy are typically switched to oral cyclosporine for no more than 12 weeks. Maintenance therapy with azathioprine is initiated at discharge, as long-term maintenance with oral cyclosporine is not recommended due to its adverse effects [74]. However, in patients who had already failed azathioprine prior to ASUC episode, initiating them on azathioprine as a maintenance therapy may not be appropriate. Instead, maintenance therapy with currently available advanced therapies following successful treatment with cyclosporin bridge therapy appears to be a logical solution in these patients. Moreover, there have been several recent reports of successful use of advanced therapies as maintenance treatment following cyclosporine rescue [75,76,77,78,79]. One of such advanced therapies is vedolizumab. In a systematic review on pooled analysis of data from observational studies, the mean response rate, defined as avoidance on colectomy, was 65% with vedolizumab and calcineurin inhibitor (cyclosporine or tacrolimus) as a bridge therapy [80]. However, the safety of this strategy should be further explored in future trials.

### 6.4. Safety of Sequential Medical Rescue Therapy

The efficacy and safety of sequential rescue therapy with a different agent when the first medical rescue therapy has failed is not clear. The major concern for this strategy is increased risk of adverse events. In a retrospective study, ten patients received infliximab after failing cyclosporin and nine received cyclosporin following infliximab. A total of 40% in the infliximab group and 33% in the cyclosporin group achieved remission. However, severe adverse events such as herpes esophagitis, pancreatitis and bacteremia were reported in the cyclosporin group, and one patient died with sepsis in the infliximab group [81] out of the 86 patients who were treated with cyclosporin and infliximab sequentially [82]. A total of 49 patients failed second-line rescue therapy, requiring colectomy, and nine patients experienced infectious complications. Moreover, one patient experienced thrombosis of pulmonary vessels after surgery. In a retrospective analysis of 47 steroid-refractory ASUC patients who received infliximab rescue therapy following failure of cyclosporin, 23% of these patients experienced adverse events and 1 patient died of hospital acquired infection [83]. Despite some success with sequential rescue therapy, its impact on long-term colectomy risk is still not clear. Therefore, sequential therapy of either infliximab followed by cyclosporine or vice versa is not recommended, and risks versus benefit should be carefully considered before opting for sequential rescue therapy.

### 6.5. Role of Unconventional Therapies in ASUC

Some non-biological therapies such as hyperbaric oxygen therapy (HOBT) and exclusive enteral nutrition (EEN) have shown to effective in limited studies. In an open-label RCT, 62 patients hospitalized with ASUC episode were randomized to receive either EEN for 7 days or standard of care. On intention to treat analysis, 25% of patients with ASUC randomized to the EEN arm failed corticosteroids compared to 43% in patients receiving standard of care. Even though this was statistically not significant, per protocol analysis showed significant difference and lack of significance was probably because of early termination of recruitment. Patients receiving the EEN arm also had a shorter hospital stay, higher day 7 albumin levels, greater reduction in serum C-reactive protein and fecal calprotectin levels compared to patients receiving standard of care. In the same study, EEN demonstrated long-term benefit in terms of lower colectomy and hospitalization rates (16% vs. 39%) at 6 months following discharge from hospital [84]. Subsequent microbiome analysis of these patients showed increased abundance of beneficial bacteria (Faecalibacterium and Veillonella) and reduced abundance of Sphingomonas with EEN, suggesting that microbial manipulation could be a possible underlying mechanism of action of EEN [85]. However, these results need to be confirmed in future studies. Another recent RCT demonstrated no additional benefit of IV albumin in patients ASUC in addition to EEN [86]. In this study, patients were randomized to two groups, in which one group received EEN and IV albumin along with corticosteroids and the control group received EEN and corticosteroids. There was no statistically significant difference between either group in steroid failure rate (33.3% vs. 41.9%). In a proof-of-concept study, patients with severe UC flare (Total Mayo score of ≥6 and Mayo endoscopic subscore of 2 or 3) requiring hospitalization were randomized in a 1:1 ratio to HBOT or sham procedure and both groups received standard of care. Only 18 patients could be included and the study was terminated early because of poor recruitment. On analysis, patients who received HOBT had higher clinical remission rates (50%) compared to patients who received sham therapy (0%). Similarly, HOBT was associated with less requirement for medical rescue therapy (10% vs. 63%, *p* = 0.04) and colectomy (0% vs. 38%, *p* = 0.07) compared to those in the sham group [87]. A major limitation of HOBT is its accessibility, and future studies with large sample sizes should be performed. In the absence of robust evidence, these newer therapies are not recommended as standard of care.

### 6.6. Role of Ultrasound Bowel Imaging in Predicting Outcomes in ASUC

Bowel ultrasound has been recently gaining interest among researchers as it is a potentially accurate tool for monitoring disease activity in UC. The distinct properties of IUS include the absence of ionizing radiation, noninvasive nature and positive acceptance by the patients. Several reports have shown that bowel ultrasound parameters such as bowel wall thickness and Doppler parameters correlate with endoscopic disease activity and accurately predict response to biologics [88,89,90]. Limited studies in ASUC also demonstrated that bowel US can be a useful tool in predicting response to IV corticosteroids. In a prospective observational study which included 10 consecutive patients with severe ulcerative colitis, bowel ultrasound was performed within 24 h of admission [91]. Colonic bowel wall thickness was significantly lower in patients who responded to corticosteroids than those who required salvage medical therapy (median thickness of 4.6 mm vs. 6.2 mm, *p* = 0.009). Authors have shown that a bowel wall thickness of >6 mm was associated with the need for salvage therapy. In another study, a significant difference between responders and non-responders was identified in both absolute bowel wall thickness [median thickness of 3.1 mm vs. 4.9 mm, *p* < 0.0001) at 48 ± 24 h [92]. A ≤20% reduction in bowel wall thickness had a sensitivity of 84.2% and a specificity of 78.4% for determining non-response (area under the curve 0.85). These findings should be replicated in large prospective studies for further validation.

## 7. Conclusions

ASUC is a potentially life-threatening relapse of UC which requires prompt recognition and admission to hospital and timely decision-making for optimal outcomes. Corticosteroids remain cornerstone to the initial management and have reduced mortality since their introduction more than 70 years ago. However, approximately one third of patients fail to respond, requiring medical rescue therapy. Delay in decision for salvage therapy is associated with worse outcomes. Therefore, timely initiation of salvage therapy by early identification of patients at risk of corticosteroid failure is crucial. A multidisciplinary team of nurses, physicians, gastroenterologists, radiologists and surgeons specialized in managing IBD should ideally be involved. Several composite models comprising clinical symptoms, biomarkers and endoscopic assessment tools have been developed for predicting response to corticosteroid therapy failure, but none are fully validated. The Oxford criteria, developed in the prebiologic era more than two decades ago, remains one of the most commonly used indices to assess response to corticosteroids. Even though some of the existing predictive indices have high specificity, their sensitivity remains poor. Newer predictive tools with high accuracy are needed for identifying patients who are likely to fail corticosteroid therapy so that medical rescue therapy can be administered upfront instead of waiting until the completion of a 5-day trial of IV corticosteroids. The potential role of newer inflammatory biomarkers such as urotensin-II in predicting steroid response should be explored [93]. A lack of predictive models for predicting the failure of medical rescue therapy is also huge unmet need. Infliximab and cyclosporine are the only agents currently recommended for medical salvage therapy. Even after successful treatment with medical rescue therapy, the risk of colectomy remains high in this group of patients. With increasing use of biologics in UC, greater numbers of patients are already exposed to anti-TNF drugs at the time of admission which might reduce efficacy of infliximab as medical rescue therapy. The efficacy of alternate medical rescue therapy such as oral JAK inhibitors needs to be explored. The efficacy of therapeutic strategies such as EEN and hyperbaric oxygen therapy should be explored in future studies.

## Figures and Tables

**Table 1:** Difference between pivotal trials in ASUC comparing Cyclosporin and infliximab for the management of steroid-refractory ASUC.

Trial	CyCIF Trial	CONSTRUCT Trial
Number of participants and centers	27 centers (France, Spain, Belgium, and Finland) From 2007 to 2010 115 (58 CsA; 57 IFX)	52 centers (England, Scotland, and Wales) From 2010 to 2013 270 (135 CsA; 135 IFX)
Participants	≥18 years Acute severe flare of UC (Lichtiger score > 10) and unsuccessful course of high-dose IV corticosteroid therapy (minimum of 0.8 mg/kg/day of methylprednisolone or equivalent for at least 5 days)	≥18 years Failed to respond to 2–5 days of IV hydrocortisone, with continuing severe disease according to Truelove and Witts’ criteria or clinical judgment
Design	Parallel, open-label, randomized controlled trial	Parallel, open-label, pragmatic randomized controlled trial This was a mixed methods trial that also evaluated cost effectiveness through a cost utility study done alongside the trial
Intervention	One-off IV IFX 5 mg/kg dose Patients who had a clinical response at day 7 received two additional infusions of 5 mg/kg IFX at days 14 and 42 and azathioprine was started on day 7 or continued in patients previously treated	IV IFX 5 mg/kg by at baseline and at weeks 2 and 6 Patients were started on azathioprine or 6-mercaptopurine at therapeutic doses in week 4 at the discretion of treating physician
Comparator	CsA by continuous IV infusion at 2 mg/kg/day Switched to oral tablets (4 mg/kg CsA in two divided doses) until day 98 in patients with clinical response at day 7	CsA by continuous infusion of 2 mg/kg per day, continued for up to 7 days followed by oral tablets (5.5 mg/kg/day two divided doses) (dose adjusted to achieve trough CsA levels of 100–200 ng/mL for 12 weeks)
Primary outcome	Treatment failure ^a^	Quality-adjusted survival
Secondary outcomes	Clinical response at day 7 Daily Lichtiger score from day 0 to day 7 Time to clinical response ^b^, Mucosal healing at day 98 (defined by a Mayo disease activity index endoscopic sub-score of 0 or 1) Quality-of-life changes from baseline to day 98 (measured with IBDQ) Colectomy free survival Safety	Change in CUCQ Change in SF-12 Change in EQ-5D
Key results	Treatment failure at day 98:60% (CsA) vs. 54% (IFX) (absolute RD 6%, 95%CI [7 to 19]) (OR 1.3, 95%CI [0.6 to 2.7]; *p* = 0.52) Clinical response at day 7:86% (CsA) vs. 84% (IFX) (absolute RD 2%, 95%CI [11 to 15]) The median time to clinical response: 5 days (IQR 4–7) (CsA) vs. 4 days (3–6) (IFX) (*p* = 0.12) Mucosal healing: 47% (CsA) vs. 45% (IFX) (absolute RD 2%, 95%CI [17 to 20]; *p* = 0.85) Severe AE: 16% (CsA) vs. 25% (IFX)	There was no significant difference in quality-adjusted survival between CsA and IFX (area under the CUCQ curve was 564.0 (SD 241.9) in the IFX group and 587.0 (226.2) in the CsA group *p* = 0.603). No difference in SF-6D scores (mean adjusted difference 0.005 [95% CI –0.025 to 0.035]; *p* = 0.737) or EQ-5D scores (QALY mean adjusted difference 0.021 [95% CI –0.032 to 0.096]; *p* = 0.350) No significant difference between allocated groups in colectomy rates: In hospital: 21% (IFX) vs. 25% (CsA) At 3 months: 29% (IFX) vs. 30% (CsA) At 12 months: 35% (IFX) vs. 45% (CsA) Overall: 41% (IFX) vs. 48% (CsA) There was no significant difference between the two drugs in serious AE

^a^ presence of any of the six criteria: absence of clinical response at day 7; relapse between day 7 and 98 [defined as a Lichtiger score increase of at least 3 points from the previous value that lasts for at least 3 consecutive days and leads to treatment modification]; absence of steroid-free remission at day 98 [defined as a Mayo disease activity index score ≤2 with an endoscopic subscore ≤1]; a severe adverse event leading to treatment interruption; colectomy; and death. ^b^ defined as the third of the first 3 consecutive days with Lichtiger response. AE: adverse events, CI: confidence interval, CsA: cyclosporine, CUCQ: Crohn’s and ulcerative colitis questionnaire, EQ-5D: EuroQol-5D, IBDQ: inflammatory bowel disease questionnaire, IFX: infliximab, QALY: quality adjusted life years, RD: risk difference, SF-12: short form-12, SF-6D: Short form-6D.
